# Cardiovascular Manifestations of Multisystem Inflammatory Syndrome in Children: A Single-Center Bulgarian Study

**DOI:** 10.3390/medicina59122175

**Published:** 2023-12-14

**Authors:** Niya Mileva, Georgi H. Vasilev, Borislav Ganev, Lyubomir Chervenkov, Hristiana Batselova, Iren Tzotcheva, Latchezar Tomov, Tsvetelina Velikova, Snezhina Lazova

**Affiliations:** 1 Medical Faculty, Medical University of Sofia, 1 Georgi Sofiiski Str., 1431 Sofia, Bulgaria; nmileva91@gmail.com; 2 Laboratory of Hematopathology and Immunology, National Specialized Hospital for Active Treatment of Hematological Diseases, “Plovdivsko pole” Str. No. 6, 1756 Sofia, Bulgaria; drgeorgivasilev@gmail.com; 3 Medical Faculty, Sofia University St. Kliment Ohridski, 1 Kozyak Str., 1407 Sofia, Bulgaria; lptomov@nbu.bg (L.T.); tsvelikova@medfac.mu-sofia.bg (T.V.); 4 Pediatric Department, University Hospital N. I. Pirogov, 21 General Eduard I. Totleben blvd, 1606 Sofia, Bulgaria; borislavganev@yahoo.com; 5 Department of Diagnostic Imaging, Medical University Plovdiv, Bul. Vasil Aprilov 15A, 4000 Plovdiv, Bulgaria; lyubo.ch@gmail.com; 6 Research Complex for Translational Neuroscience, Medical University of Plovdiv, Bul. Vasil Aprilov 15A, 4002 Plovdiv, Bulgaria; 7 Department of Epidemiology and Disaster Medicine, Medical University of Plovdiv, University Hospital “St George”, blvd. Vasil Aprilov 15A, 4000 Plovdiv, Bulgaria; dr_batselova@abv.bg; 8 Pediatric Clinic, UMHATEM “N. I. Pirogov”, Blvd. “General Eduard I. Totleben” 21, Pette Kyosheta, 1606 Sofia, Bulgaria; irenmd@yahoo.com; 9 Department of Informatics, New Bulgarian University, Montevideo 21 Str., 1618 Sofia, Bulgaria; 10 Department of Healthcare, Faculty of Public Health “Prof. Tsekomir Vodenicharov, MD, DSc”, Medical University of Sofia, Bialo More 8 Str., 1527 Sofia, Bulgaria

**Keywords:** multisystem inflammatory syndrome in children, MIS-C, COVID-19, SARS-CoV-2 infection, cardiovascular involvement

## Abstract

*Background and objectives*: Multisystem inflammatory syndrome in children (MIS-C) poses challenges to the healthcare system, especially with frequent heart involvement. The current retrospective observational study aims to summarize the type and degree of cardiovascular involvement in children with MISC and to find possible associations between laboratory, inflammatory, and imaging abnormalities and the predominant clinical phenotype using a cluster analysis. *Material and methods:* We present a retrospective observational single-center study including 51 children meeting the MIS-C criteria. *Results*: Fifty-three percent of subjects presented with at least one sign of cardiovascular involvement (i.e., arterial hypotension, heart failure, pericardial effusion, myocardial dysfunction, pericarditis without effusion, myocarditis, coronaritis, palpitations, and ECG abnormalities). Acute pericarditis was found in 30/41 of the children (73%) assessed using imaging: 14/30 (46.7%) with small pericardial effusion and 16/30 (53.3%) without pericardial effusion. The levels of CRP were significantly elevated in the children with pericarditis (21.6 ± 13 mg/dL vs. 13.9 ± 11 mg/dL, *p* = 0.035), and the serum levels of IL-6 were higher in the children with small pericardial effusion compared to those without (191 ± 53 ng/L vs. 88 ± 27 ng/L, *p* = 0.041). Pericarditis with detectable pericardial effusion was significantly more frequent in the female vs. male subjects, 72% vs. 30% (*p* = 0.007). The hierarchical clustering analysis showed two clusters: Cluster 1 includes the children without cardiovascular symptoms, and Cluster 2 generalizes the MIS-C children with mild and severe cardiovascular involvement, combining pericarditis, myocarditis, heart failure, and low blood pressure. Also, subjects from Cluster 2 displayed significantly elevated levels of fibrinogen (5.7 ± 0.3 vs. 4.6 ± 0.3, *p* = 0.03) and IL-6 (158 ± 36 ng/mL vs. 66 ± 22 ng/mL, *p* = 0.032), inflammatory markers suggestive of a cytokine storm. *Conclusions*: Our results confirm that children with oligosymptomatic MIS-C or those suspected of long COVID-19 should be screened for possible cardiological involvement.

## 1. Introduction

The SARS-CoV-2 coronavirus caused the COVID-19 pandemic in 2020, resulting in widespread morbidity and mortality affecting almost every nation globally [1]. Although most infected children do not develop severe disease, an unusual pediatric syndrome called multisystem inflammatory syndrome in children (MIS-C) is thought to be associated with previous infection with SARS-CoV-2. This poses additional challenges to the healthcare system and public health. In line with this, by the end of June 2020 alone, approximately 1000 cases of MIS-C had been reported worldwide [1].

Children diagnosed with MIS-C can have very different clinical manifestations, and the course of the disease varies in severity [2]. Therefore, MIS-C is a newly recognized, rare, pediatric hyperinflammatory disorder that affects children several weeks after infection with SARS-CoV-2 (COVID-19) [3], most commonly occurring within two to four weeks of infection with SARS-CoV-2 [4,5]. However, some children with MIS-C have no prior symptoms of COVID-19 [2,6]. Demonstrating that infection with SARS-CoV-2 can lead to serious medical consequences in children, such as MIS-C [7], emphasizes the increasing recognition of the syndrome and its significance in the pediatric population. SARS-CoV-2 viral structures and the host immune response have been suggested to lead to hyperinflammatory syndromes in children (MIS-C) and adults [8,9,10,11,12]. The first epidemiological study of MIS-C in Europe showed a cumulative incidence of 3.27 per 100,000 inhabitants and a prevalence of 74 cases per 100,000 pediatric subjects with previous SARS-CoV-2, between 0 and 18 years of age. A higher incidence of gastrointestinal and cardiac involvement has been demonstrated with myocarditis compared with Kawasaki disease [13].

The condition can develop in previously healthy children without comorbidities [14]. However, according to Canadian scientists, children with at least one comorbidity are more likely to develop MIS-C [15]. There is evidence that MIS-C can begin with very different symptoms, ranging from persistent fever and some features of Kawasaki disease to the most severe shock and multiorgan failure [16]. Other researchers, including us, have reported that the most common symptoms of MIS-C are fever, gastrointestinal symptoms (abdominal pain, vomiting, and diarrhea), rash, and conjunctivitis [17,18,19,20].

Cardiovascular manifestations in MIS-C are common, occurring in 34−82% of cases. They include myocardial dysfunction due to microangiopathy, myocarditis, coronary artery dilation or aneurysms, conduction abnormalities, arrhythmias, pericarditis, and valvulitis. Severe cases can present with distributive or cardiogenic shock requiring fluid resuscitation and inotropic support. Among the others, left ventricular (LV) systolic dysfunction is the most frequent abnormal cardiac finding [21].

Since cardiac symptoms are common in MIS-C, it is essential to investigate further in all patients. The potential cardiac complications could be life-threatening; therefore, patient care is mainly focused on early diagnosis and patient stabilization. Notwithstanding, with data accumulation, many improvements in diagnosis, treatment, and long-term care for children with MIS-C have been documented [22].

However, despite numerous studies on risk factors and clinical prognosis, many unknowns remain regarding the causal relationship, optimal prevention and treatment interventions, and long-term outcomes in patients with MIS-C [16]. Moreover, the lack of definitive data on the risks of developing MIS-C in SARS-CoV-2-infected children is an additional challenge during the COVID-19 pandemic [23].

The aims of the current study are to analyze the rate of cardiovascular involvement in children with MIS-C, describe the specific type and degree of cardiovascular impairment, and assess possible associations between laboratory and imaging findings and the predominant clinical phenotype.

## 2. Materials and Methods

### 2.1. Design of the Study

**Figure 1:**
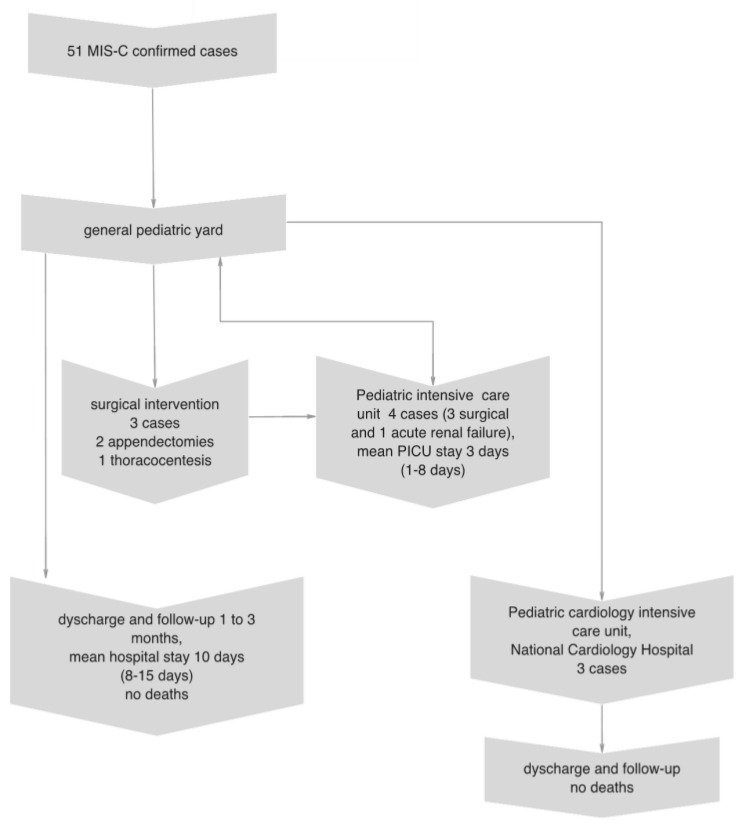
Flow chart of the children with multisystem inflammatory disease included in the study.

We present a retrospective observational single-center study. Most of the enrolled children were initially hospitalized in the general pediatric yard. One child with symptoms of acute renal failure at admission was referred directly to the PICU of the hospital. After the first 48–72 h, the conditions of four children worsened, with the need for a PICU transfer. Three underwent surgery (two adenectomies and one thoracocentesis), and one required non-invasive respiratory support and inotropic medication. Three children with rapidly progressing myocarditis and heart failure were transferred to the pediatric cardiology intensive care unit in the National Cardiology Hospital. There were no deaths, and the enrolled children were followed up for 1 to 6 months (Figure 1).

### 2.2. Subjects

For the period of 25 November 2020 to 24 April 2021, 51 children meeting the MIS-C diagnostic criteria according to the CDC criteria for an MIS-C case (Centers for Disease Control and Prevention. Case Definition for MIS-C. 2020. Available online: https://cdn.ymaws.com/www.cste.org/resource/resmgr/ps/ps2022/22-ID-02_MISC.pdf (accessed on 12 December 2022), WHO, and the Royal College of Pediatrics and Child Health (RCPCH, UK) [9], were referred, admitted, and followed up at the Pediatric Clinic of the University Emergency Hospital Pirogov in Sofia, Bulgaria. All the children were anti-SARS-CoV-2 seropositive, and three had positive rapid antigen and/or PCR tests of the nasopharyngeal swab at their admission or repeated assessment [18,19].

The children that met the eligibility criteria for case definitions of MIS-C were included in the study and defined as the MIS-C group [19] as follows:

- Age under 18 with fever (>38.0 °C for ≥24 h or subjective fever lasting ≥24 h), inflammation (laboratory-confirmed by abnormal C-reactive protein (CRP), erythrocyte sedimentation rate (ESR), fibrinogen, procalcitonin (PCT), D-dimer, ferritin, lactic acid dehydrogenase (LDH), or interleukin 6 (IL-6), higher neutrophils, low lymphocytes, and low albumin), and clinically severe COVID-19 requiring hospitalization, presenting with multisystem (>2) organ involvement; AND
- Other diagnoses excluded; AND
- RT-PCR, antigen, or serological tests proving recent or current infection with SARS-CoV-2, or exposure to the virus four weeks ago.

Fourteen (27.5%) of the MIS-C patients had the following comorbidities: drug allergies, allergic rhinitis, mild bronchial asthma, cerebral palsy, and epilepsy.

The children were treated according to the local protocol with broad-spectrum antibiotics and systemic corticosteroids. In the indicated cases, human serum albumin (27 children), intravenous immunoglobulins (13 children), anticoagulants—enoxaparin (38 children), ASA (12 children), diuretics (i.e., furosemide 25 children), inotropic support (5 children), oxygen therapy (17 children), and nasal CPAP (1 child) were administered. At that moment, in our department, no biological treatment was available.

### 2.3. Clinical Methods

A complete medical history (including epidemiological history and data for any concomitant diseases or conditions) and physical examination by a pediatrician and a pediatric surgeon in the indicated patients were taken and performed at admission. Anthropometric measurements were also taken, and the children’s body surface and BMI were calculated. In all the children, continuing vital sign monitoring and ECG (electrocardiography) records were performed during the first 48 h and longer when needed.

### 2.4. Laboratory Workup

#### 2.4.1. General Laboratory Assessment

At admission and during the hospital stay, when indicated, the following laboratory blood tests were performed: complete blood count, erythrocyte sedimentation rates, inflammatory markers (C-reactive protein (CRP), procalcitonin (PCT)), ferritin, AST (aspartate aminotransferase, SGOT), ALT (SGPT, alanine aminotransferase), GGT (gamma-glutamyl transferase), LDH (lactate dehydrogenase), ALP (phosphatase, alkaline), total serum proteins and albumin, total and direct bilirubin, coagulation INR (international normalized ratio), PT (prothrombin time), d-dimer, and fibrinogen). CPK (creatine phosphokinase), the CK-MB fraction (creatine kinase-MB fraction), and troponin I were assessed in the cases with markedly increased inflammatory markers [19] and clinical suspicion of cardiologic involvement.

#### 2.4.2. Immunological Assessment

A serological evaluation of anti-SARS-CoV-2 antibodies was performed in fifty of the children using two laboratory methods: quantitative total IgM and IgG in 14 of the children and qualitative IgG titer in the other 36. Interleukin-6 (IL-6) was assessed in 32 of the children as an inflammatory marker suggestive of a cytokine storm. A certified immunological laboratory performed all the immunological tests.

### 2.5. Imaging Testing

The following imaging studies were undertaken: chest and abdominal radiographs; computed tomography (CT) scans of the chest, abdomen, and pelvis; thoracic, abdominal, neck, and testicular ultrasound (US), and echocardiography (EchoCG). An abdominal US was performed in all the cases, and the other imaging modalities were performed when indicated [24].

An experienced pediatric cardiologist conducted an EchoCG on 36 of the patients. The assessment included a transthoracic//TTE/two-dimensional (2D) echocardiography, M-mode echocardiography, and Doppler echocardiography. The M-mode Teichholz and two-dimensional Sympson’s biplane methods were used to assess the cardiac systolic function (left ventricular ejection fraction—LVEF). The mean values taken from three consecutive heart cycles were considered. Fractional shortening (FS) was based on the M-mode and categorized as either a normal (25–43%), mild (20–24%), moderate (15–19%), or severe reduction (≤14%). The left ventricular ejection fraction (LVEF) was based on the modified Simpson’s method and categorized as either a normal (≥55%), mild (45–54%), moderate (30–44%), or severe impairment (<30%). The valvular function was assessed based on the pulse, continuous wave, and color Doppler. In five of the children, assessing the coronary vessel dimensions was technically possible. Coronary artery z-scores were derived from previously described normative data (Boston Z score system) and used to classify coronary artery abnormalities as follows: normal: <2, dilatation: 2 to <2.5, and aneurysm: >2.5.

The pericardial involvement, including the pericardial reaction with or without pericardial effusion, was defined using ECHO and CT scans where applicable.

### 2.6. Statistical Analysis

Statistical analysis and graphical processing were conducted using SPSS version 29 (2023), Jupyter Notebook 7.0.6 (Python 3.11.6), R-Studio (Version 2023.06.3+581), and GraphPad Prism 6. We performed descriptive statistics, Kolmogorov–Smirnoff tests, T-tests, Mann–Whitney tests, ANOVAs, χ^2^ or Fisher’s Exact tests, IndependVent samples proportion Z test correlation analysis, hierarchical cluster analysis, K-means cluster analysis, factorial analysis of mixed data (FAMD), and uniform manifold approximation and projection for dimension reduction (UMAP). *p* values < 0.05 were considered significant.

This study’s principal investigators collected data according to the hospital’s ethical and other policies and good clinical practice. The data were collected and coded uniformly to avoid any potential sources of bias. Efforts were made to address potential bias. The statistical analyses were performed by omitting empty entries for missing data (but no more than 5% of all the data entries). No sensitivity analyses were performed in our study.

### 2.7. Ethics

This study was conducted following the Declaration of Helsinki and the Ethics Committee of the University Hospital. N. I. Pirogov approved the study design and protocol (No 123-20/23.12.2020). All the parents signed informed consent for the inclusion of their children in the study. Additionally, all the children older than 12 years signed informed consent on their own before participating in the study, in addition to the signed consent from their parents.

## 3. Results

### 3.1. Demographic and COVID-19 Exposure Status

For the study purposes, 51 children were consecutively recruited, including 73% (N = 37) boys and 27% (N = 14) girls, with statistically significant male preponderance (binominal one-sample proportion test, Z = 4.06, *p* = 0.001) and children older than five years of age, 78% (N = 40) vs. 22% (N = 11), *p* = 0.001 (binominal one-sample proportion test, Z = 3.22).

Regarding the COVID-19 exposure status, 29% (N = 15) of the children had a positive epidemiology history of COVID-19 exposure in the past month (contact with a family member), and 41% (N = 21) had a positive history of COVID-19 symptoms. Children with a positive history of COVID-19-related symptoms had significantly increased D-dimer levels compared to those without, 2912 ± 545 ngFEU/mL vs. 1833 ± 334 (exact Mann–Whitney U-test, *p* = 0.05). No significant differences in the proportion of children with pericarditis, myocarditis, and signs of cardiovascular involvement were observed between the two age groups: younger and older than five years (independent samples proportion Z test, *p* > 0.05). The distributions of demographic characteristics, general symptoms, and heart involvement are shown in Table 1 and Table 2.

### 3.2. Cardiovascular Symptoms

#### 3.2.1. Pericarditis and Pericardial Effusion

**Figure 2:**
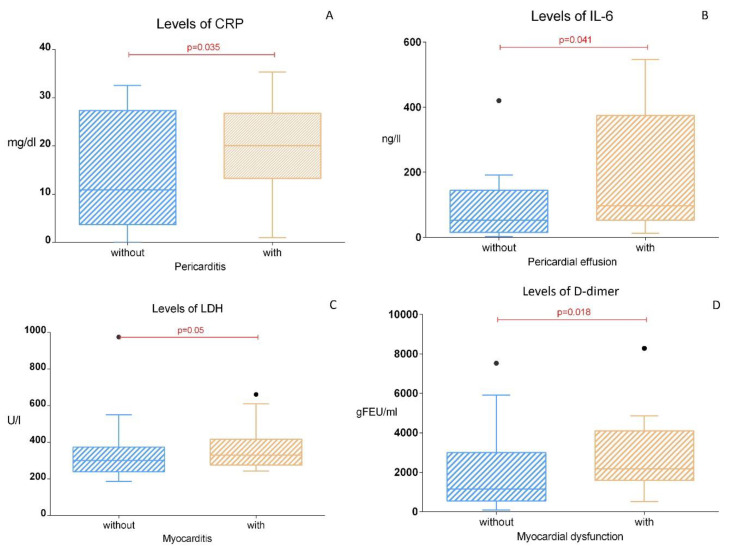
Levels of CRP depending on the presence of pericarditis (**A**), levels of IL-6 depending on the presence of pericardial effusion (**B**), levels of LDH depending on the presence of myocarditis (**C**), and levels of D-dimer depending on the presence of myocardial dysfunction (**D**).

Of the 42 children, 36 had an echocardiography, and 4 underwent CT scans for the evaluation of pericarditis/pericardial effusions. Based on the echoCG results, 73% (N = 30/41) presented with ultrasonic evidence of acute pericarditis. Fifty-three percent (N = 16) of the children with pericarditis had a small pericardial effusion, and 46.7% (N = 14) had pericarditis (thickening and hyperechogenicity of the pericardial layers) without detectable pericardial effusion. The presence of pericarditis was positively associated with signs of cardiovascular involvement at 83% (N = 25) vs. 17% (N = 2), *p* = 0.001 (independent-samples proportions test, Z = 4.1). The levels of CRP were significantly elevated in the children with pericarditis, 21.6 ± 13 mg/dL vs. 13.9 ±11 mg/dL, *p* = 0.035 (Figure 2A). We observed significantly elevated serum levels of IL-6 in the children with small pericardial effusions compared to those without, 191 ± 53 ng/L vs. 88 ± 27 ng/L, *p* = 0.041 (Figure 2B). Pericarditis with detectable pericardial effusion was significantly more frequent in female vs. male subjects, 72% (N = 8 out of 11 females) vs. 30% (N = 8 out of 27 males), *p* = 0.007.

#### 3.2.2. Myocarditis

Regarding myocarditis, only 11% (N = 6) of the recruited MISC subjects presented with clinical, laboratory, and echocardiographic evidence of myocarditis. The children with myocarditis had significantly elevated levels of Troponin and LDH compared to those without myocarditis, 286 ± 110 vs. 57 ± 30 pg/mL (*p* = 0.08) (Figure 2C) and 392 ± 55 vs. 356 ± 37 U/L (*p* = 0.05) (Figure 2D). All the patients with myocarditis also presented with other cardiovascular signs (Fisher exact test *p* = 0.04) and echocardiographic signs of myocardial dysfunction (Fisher exact test *p* = 0.008). No gender-related differences were observed.

**Figure 3:**
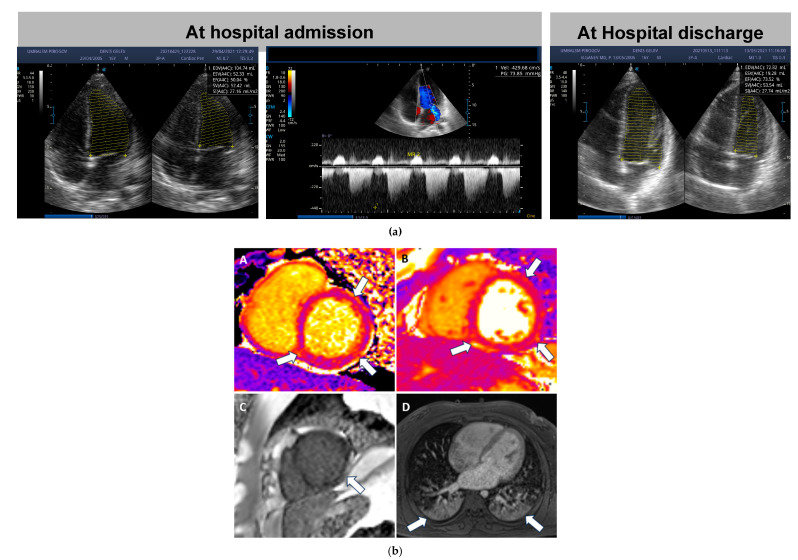
**Diagnostic imaging of myocardial involvement in MIS-C.** (**a**) EchoCG assessment of a 16-year-old boy with low-flow heart failure presenting with arterial hypotension. EchoCG data for moderate myocardial dysfunction with dilatation of the chambers and valvular insufficiency at admission (LVDd, mm—38.7, LVDs, mm—51.6, LVSF—28.7% LVEF 48.7%) and after hospital discharge (LVDd, mm—17.8, LVDs, mm—40.4, LVSF—52% LVEF 82.6%). (**b**) Cardiac magnetic resonance: T1 (**A**) and T2 (**B**) mapping show diffusely elevated relaxation times seen as areas in lighter orange color consistent with edema. Late gadolinium enhancement sequence (**C**) in apical short axis view shows subendocardial enhancement in inferolateral apical wall involving up to 75% of the myocardial thickness. Bilateral lung consolidations are present (**D**).

In Figure 3, we present echoCG data of a 16-year-old male patient with myocarditis (a) and Cardiac magnetic resonance (CMR) data of myocardial involvement/myocarditis in another 16-year-old male patient (b) from our MIS-C cohort.

The cardiac magnetic resonance (CMR) reveals biventrical dilatation with diffuse hypokinesia and reduced left ventricular ejection fraction–45%. There are patchy high signals on T2 TIRM images in virtually all segments of the left chamber and right ventricle apex. T1 and T2 relaxation times are diffusely elevated–up to 1200 ms for T1 (normal range under 1100 ms) and up to 60 ms for T2 (normal range under 50 ms). Late gadolinium enhancement sequence shows subendocardial enhancement engaging up to 75% of myocardial thickness in inferolateral apical wall and diffusely patchy midwall and subepicardial enhancement. Bilateral areas of consolidations were present in dorsobasal lung segments. (Figure 3b). 

The MRI finding are consistent with acute myocarditis with areas of edema, identifiable as high signals on T2 TIRM images and elevation of T1 and T2 relaxation times as well as non ischemic late gadolinium enhancement corresponding to myocardial necrosis/fibrosis. The subendocardial late enhancement correspond to small myocardial infarction.

#### 3.2.3. Blood Pressure

Regarding the blood pressure levels in our MISC cohort, only 15% (N = 8) of the study subjects had high blood pressure according to age, 36% (N = 18) presented with hypotension, and the remaining 49% (N = 25) had no blood pressure abnormalities. Regarding blood pressure and gender, all the subjects with high blood pressure were males, *p* = 0.05. Using the chi-square test, we obtained a positive association between low blood pressure levels and myocardial dysfunction, Fisher exact test, *p* = 0.04.

#### 3.2.4. Signs of Cardiovascular Involvement

A total of 27/51 of the children (53%) presented with at least one sign of cardiovascular involvement (arterial hypotension, heart failure, pericardial effusion, myocardial dysfunction, pericarditis without effusion, myocarditis, coronaritis, palpitations, ECG abnormalities). None of the children had pericardial chest pain and detectable pericardial rubs upon auscultation. Their cardiovascular symptoms (heart involvement) correlated positively with myocardial dysfunction (Cramer’s V = 0.43, *p* = 0.05). All the children with echocardiographic data for septal dyskinesia had at least one cardiovascular involvement sign—60% (N = 11) vs. 0% (N = 0), independent samples proportion Z-test, *p* = 0.003.

**Figure 4:**
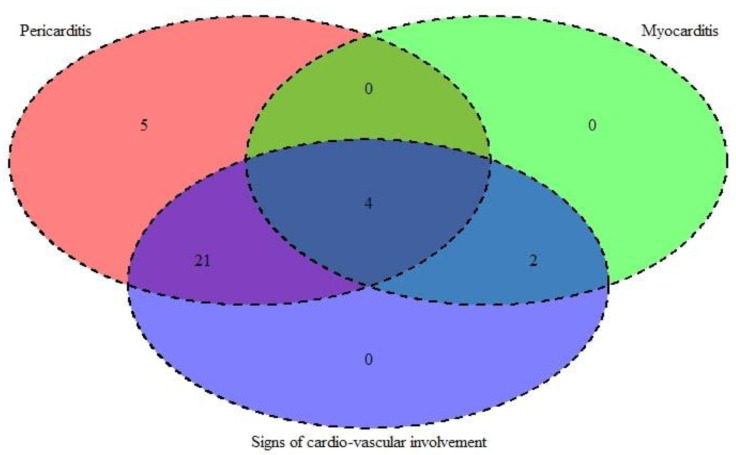
Venn diagram displaying the co-presentation of pericarditis, myocarditis, and signs of cardiovascular involvement in our MIS-C study cohort.

In Figure 4, we present the co-presentation of pericarditis, myocarditis, and signs of cardiovascular involvement in the MIS-C patients.

#### 3.2.5. Myocardial Dysfunction, Septal Dyskinesia, Heart Wall Dyskinesia, Coronary Artery Dilatation

The echocardiographic characteristics of the investigated children with MIS-C are presented in Table 3.

In 10 of the children, there were echocardiographic data for heart wall dyskinesia. Six of them had antero-septal localization, one had antero-lateral localization, eleven had septal dyskinesia, and nine had co-existing heart wall dyskinesia. In three of the children, we found evidence of left ventricular dysfunction with a left ventricular ejection fraction (LVEF) of less than 50%, and one child had a LVEF of 33.2%. Slight coronary artery dilatation was found in four of the children—two with slight left coronary artery dilatation, one with an affected common coronary artery, and one with an affected right coronary artery. None of the children had coronary aneurysms at the initial and follow-up examinations. A strong association was found between myocardial dysfunction, septal dyskinesia (Cramer’s V = 0.59, *p* = 0.008), and heart wall dyskinesia (Cramer’s V = 0.52, *p* = 0.03). The MISC subjects with myocardial dysfunction displayed significantly higher levels of D-dimer as a marker of macrophage activation, 3397 ± 858 ngFEU/mL vs. 1975 ± 491 ngFEU/mL, *p* = 0.018 (Figure 2D). Septal dyskinesia was linked more often with pericarditis (likelihood ratio test, *p* = 0.034) and heart wall dyskinesia (likelihood ratio test, *p* = 0.001). A strong association was also found between septal dyskinesia and anteroseptal heart wall dyskinesia, Cramer’s V = 0.8, *p* = 0.001. We used a chi-square test to find a significant association between heart wall dyskinesia, symptoms of cardiovascular involvement (*p* = 0.016), and septal dyskinesia (*p* = 0.001). The predominant localization of LV wall dyskinesia was antero-septal, *p* = 0.002.

### 3.3. Clustering and Dimensionality Reduction Techniques for Mixed Data

**Figure 5:**
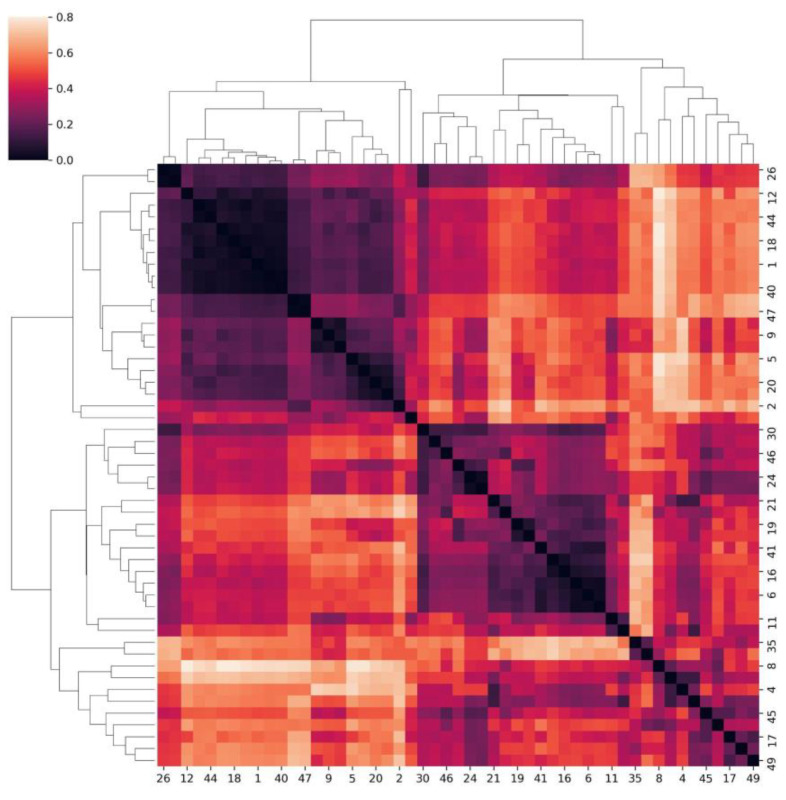
Heatmap and dendrogram from the hierarchical cluster analysis displaying the individual Gower’s distances.

Cluster analysis and dimensionality reduction techniques were applied to identify underlying patterns in the data structure and to analyze the complex clinical presentation of different symptoms and cardiovascular involvement in a more sophisticated and easy-to-comprehend visual manner. Since both numerical (fibrinogen, troponin, D-dimer) and categorical variables (pericarditis, pericardial effusion, myocarditis, myocardial dysfunction, heart failure, blood pressure levels) were used for the cluster analysis, firstly, a Gower distance matrix was calculated based on the Gower’s distance (the “gower” python package was used). A kernel principal component analysis (KPCA) was conducted based on the individual distances. Furthermore, hierarchical clustering analysis using the “Ward” method and K-means analysis on each patient’s score on the first two components of the KPCA were applied. For visualization purposes, a heatmap with a dendrogram displaying the Gower matrix and the hierarchical cluster analysis was produced (Figure 5). From the heat map, we can easily observe that two significant clusters can be identified, which agrees with the results obtained using the elbow method applied to determine the optimal cluster number in our data set.

**Figure 6:**
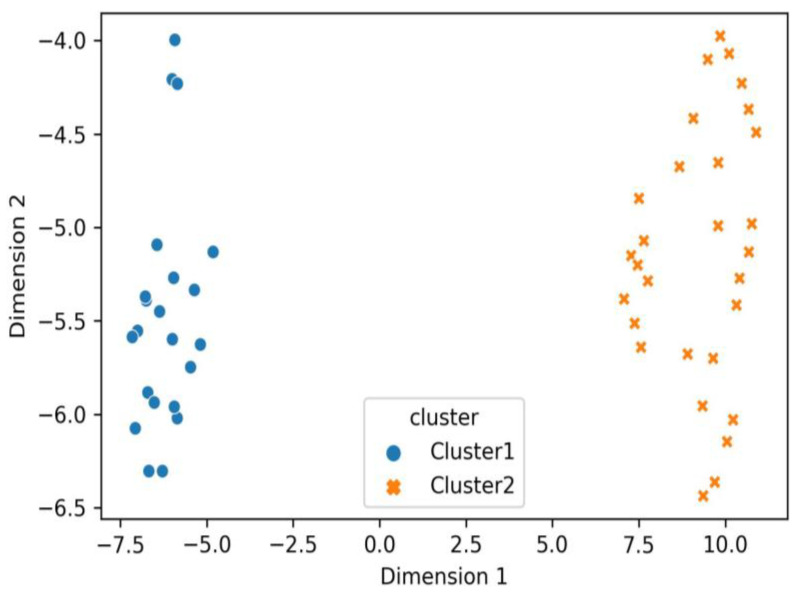
Scatter plot displaying the distribution of the patients from both clusters on the UMAP Dimension 1 and UMAP Dimension 2.

**Figure 7:**
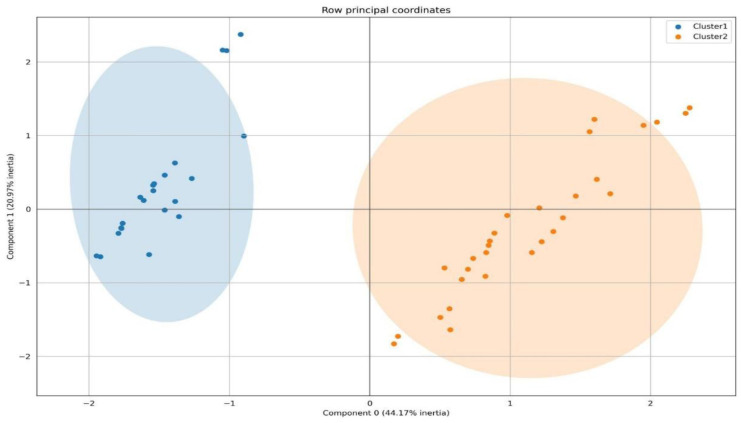
Individual score plot generated using FAMD. All the MIS-C subjects are presented in the plot. Cluster 1 subjects are depicted in blue and Cluster 2 subjects are depicted in orange. The FAMD plot generalized 65% of all inertia.

As dimensionality reduction and data visualization methods, we applied two techniques: factorial analysis of mixed data (FAMD) and uniform manifold approximation and projection for dimension reduction (UMAP). The results are shown in Figure 6 and Figure 7.

**Figure 8:**
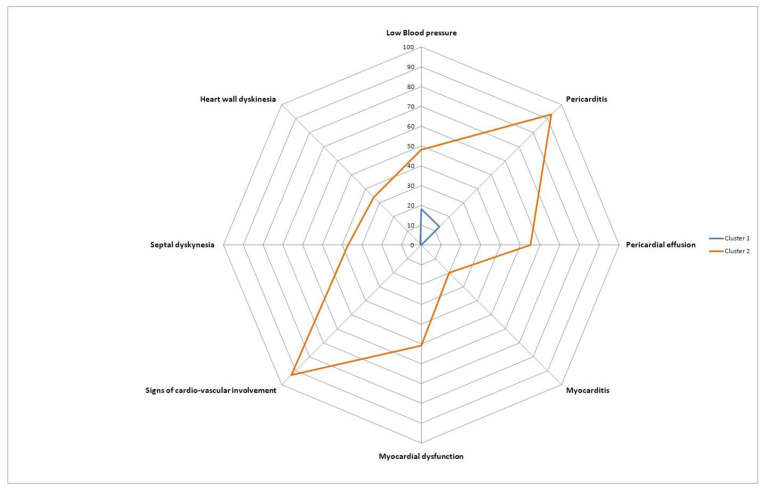
Radar plot displaying the involvement of the cardiovascular system across Cluster 1 and 2 patients, expressed as the percentage of patients positive for different features depicted on the radar plot axes.

Figure 6 and Figure 7 provide visualizations of the two patient clusters for the purposes of explanatory data analysis. We can see that both clusters are entirely separable and thus generalize the combined presentation of clinical, ultrasonographic, and clinical parameters regarding cardiovascular involvement. For diligence, one of the two clusters was termed Cluster 1, including 43% (N = 22) of the subjects. The other cluster, Cluster 2, contained 57% (N = 29) of the study subjects enrolled (Figure 8). No gender and age-related differences were observed between the clusters. Regarding blood pressure, Cluster 2 was associated with a lower for age blood pressure levels, *p* = 0.05. In Cluster 2, patients with pericarditis (93% of Cluster 2 and 90% of all pericarditis patients, *p* = 0.001), cardiovascular symptoms (100% of Cluster 2 patients and 100% of all patients with cardiovascular symptoms, *p* = 0.001), myocardial dysfunction (*p* = 0.011), pericardial effusion (*p* = 0.001), and septal and heart wall dyskinesia (*p* = 0.002) predominated. Thus, Cluster 1 included children without cardiovascular symptoms. Cluster 2 generalized the MIS-C children with mild and severe cardiovascular involvement, combining pericarditis, myocarditis, heart failure, and low blood pressure. Also, subjects from Cluster 2 displayed elevated levels of fibrinogen (5.7 ± 0.3 vs. 4.6 ± 0.3, *p* = 0.03) and IL-6 (158 ± 36 ng/mL vs. 66 ± 22 ng/mL, *p* = 0.032), inflammatory markers suggestive of a cytokine storm.

Additional analysis revealed a correlation between pericardial effusion and IL-6 levels (*p* = 0.27), D-dimer (*p* < 0.001), and PCT (*p* = 0.049). The patients with pericardial effusions presented with elevated levels of IL-6 (168.52 vs. 67.53 ng/mL), D-dimer (2925.13 vs. 1207.69 ngFEU/mL), and PCT (11.58 vs. 5.54 ng/mL) compared to those without pericardial effusions, respectively. No significant differences were observed in the levels of fibrinogen and CRP regarding pericardial effusions. For ascites, we found higher levels of PCT in children with ascites compared to those without (8.52 vs. 7.1, *p* = 0.024), as well as a tendency of presenting with higher D-dimer (2636.77 vs. 1624 ngFEU/mL, *p* = 0.093).

The distributions of the mentioned laboratory markers in the two clusters are presented in Table 4.

We found that Cluster 1 and Cluster 2 differed significantly regarding their IL-6 and fibrinogen levels. Most of the other markers were elevated in Cluster 2 (except albumin, which was reduced), although without reaching statistical significance.

## 4. Discussion

In this retrospective observational study, we aimed to summarize the type and degree of cardiovascular involvement in children with MISC and to find possible associations between the laboratory, inflammatory, and imaging abnormalities and the predominant clinical phenotype using a cluster analysis.

We found that more than half of the study subjects (53%) presented with at least one sign of cardiovascular involvement (i.e., arterial hypotension, heart failure, pericardial effusion, myocardial dysfunction, pericarditis without effusion, myocarditis, coronaritis, palpitations, ECG abnormalities). Other studies have demonstrated cardiac involvement in 67–80% of MIS-C patients [25,26,27,28,29,30,31].

We must admit that the leading symptoms in our cohort of MIS-C patients were gastrointestinal. This may have been influenced by the fact that our hospital is a referral center for pediatric abdominal and thoracic surgery. On the other hand, specialized pediatric cardiology medicine is the focus of another center (the National Cardiology Hospital), and cases with leading cardiology symptoms are referred directly there.

Cardiovascular involvement in MIS-C may range from mild to severe (i.e., heart failure and cardiogenic shock). The percentage of cardiovascular involvement in our cohort of patients may be explained by the fact that patients with leading cardiovascular complications would be treated directly without investigating the possibility of a MIS-C diagnosis.

Our results demonstrate acute pericarditis in 71.4% of the patients with cardiovascular symptoms, small pericardial effusion in 38% of the patients, and pericarditis in 33% of them. In the literature, ventricular dysfunction was found in 33–50% of children with MIS-C [25,32,33,34].

Clinically, ventricular dysfunction usually involves myocarditis, or it results from generalized inflammation or loading changes [21]. Ventricular dysfunction can be evaluated using echocardiography and/or cardiac MRI (CMR) [33,35,36].

We performed a CMR on a 16-year-old boy with myocarditis who had comorbidities (concomitant renal and hepatic dysfunction) and presented with a clinic picture of pronounced, low-output heart failure with arterial hypotension. In this case, acute myocarditis was established using MRI, and the link with SARS-CoV-2 was based on the history of possible COVID-19 and the presence of elevated IgM and IgG antibodies against the virus. MRI plays an essential role in the diagnosis of multisystem inflammatory syndrome. In studies of patients with this syndrome, hyperintensity of the left ventricle has been detected using MRI, which is associated with interstitial edema [27]. In a magnetic resonance imaging study of 18 children with an average age of 12 years who were diagnosed with COVID-19, no structural changes associated with myocarditis were found. Small pericardial effusion was found in 17% of the patients [37]. Another study was conducted on 20 children with an average age of 12 years with a known history of COVID-19. They underwent a cardiac MRI, which revealed normal volume and function of the cardiac chambers in 80% of the patients and a reduced LV ejection fraction in the remaining 20%. In MIS-C, the heart is often involved, with ventricular dysfunction, arrhythmia, and pericardial effusions being the most common findings [38].

CMR can reveal T1 and T2 abnormalities in the acute phase; however, several studies have proposed CMR to evaluate MIS-C patients after the acute phase (2–8 weeks after the illness) [27,32,33,39,40]. CMR can also detect strain abnormalities in MIS-C patients with reduced LVEF, including in T2 (0–33%) and T1 (0–5% of MIS-C patients) images [39].

However, there are some limitations to using MRI in children, such as clinical instability and the need for anesthesia in smaller children [21]. Recently, we demonstrated the significance of abdominal and thoracic imaging for evaluating gastrointestinal complications in children with MIS-C [24]. Additionally, limited autopsies have shown heart injuries in deceased MIS-C patients, such as inflamed endo-, myo-, and pericardium, along with contraction band necrosis [41,42].

We obtained a positive association between low blood pressure levels and myocardial dysfunction. In line with this, considering that the main symptom of myocardial dysfunction is hypotension, we suggest that all children with hypotension and anamnestic data for COVID-19 be assessed for cardiac manifestation in MIS-C. Additionally, we found that MISC subjects with myocardial dysfunction displayed significantly higher levels of D-dimer, which could be a marker of macrophage activation and is a possible risk factor for uncontrolled systemic inflammation.

Our patients with myocardial dysfunction, as reported by other investigators, fully recovered on the follow-up at the 1st, 3rd, and 6th month after hospital discharge [25,34]. Echocardiography is essential in assessing systolic and diastolic dysfunction in MIS-C patients [43]. A reduced left ventricular ejection fraction is reported most commonly (in 34–50% of MIS-C cases) [25,28,32,33,36,39,43].

We also found a LVEF of <50% in three of our MIS-C patients, one with 33.2%. Additionally, abnormal ventricular strain in MIS-C patients could be observed [35,43].

Furthermore, some studies have demonstrated that lower strain values are more frequent than a reduced ejection fraction [36]; however, decreased strain values are usually associated with worse clinical outcomes and prognoses [35,36]. We did not assess the strain values in our patients, but we found that the predominant localization of LV wall dyskinesia was antero-septal.

In the patients with pericarditis, the serum levels of CRP were elevated significantly, and IL-6 was increased dramatically in our cohort of MIS-C children with small pericardial effusion compared to those without. This inflammatory activity is the object of the immunomodulatory approach of treating MIS-C with corticosteroids, IVIG, and biologics (i.e., anti-IL-6, etc.) [38]. However, none of our patients received biologics. Still, all were treated with supportive therapy, and some of them were treated with corticosteroids and anti-thrombotic agents (because of the risk of thrombotic complications and increased mortality [44,45]).

Especially valid is combined therapy with anticoagulants +/− antiplatelets for the prevention of intracardiac thrombi and embolic events in MIS-C patients with mild to moderate depression of ventricular function [46].

Interestingly, pericarditis with detectable pericardial effusion was significantly more frequent in female vs. male subjects (72% vs. 30%) in our cohort of patients. This finding showed that all the MIS-C female patients in our study presented with pericarditis with or without pericardial effusions, and pericardial effusions were more frequent in female than male patients. We had only one case with ECG abnormalities in our MIS-C cohort (approximately 2%), which was lower than the rate of arrhythmias and conduction abnormalities visible on ECGs (28–67%) [47,48,49].

Our investigation also included hierarchical clustering analysis using the “Ward” method and K-means analysis on each patient’s score on the first two components of the kernel principal component analysis, which we believe is a solid contribution to cardiovascular involvement in MIS-C. We fund two distinct clusters. Cluster 1 included children without cardiovascular symptoms, and Cluster 2 generalized the MIS-C children with mild and severe cardiovascular involvement, combining pericarditis, myocarditis, heart failure, and low blood pressure. Also, the subjects in Cluster 2 displayed significantly elevated levels of fibrinogen (5.7 ± 0.3 vs. 4.6 ± 0.3, *p* = 0.03) and IL-6 (158 ± 36 ng/mL vs. 66 ± 22 ng/mL, *p* = 0.032), inflammatory markers suggestive of a cytokine storm.

Coronary artery abnormalities are other common observations in MIS-C. Moreover, although MIS-C is distinct from Kawasaki disease, both conditions share some similarities, such as coronaritis and coronary artery dilation [7]. We observed four cases (11%) of coronaritis in our cohort of MIS-C patients, but no other abnormalities. The prevalence of coronary artery aneurysms in MIS-C reported in the literature is between 13–26% [25,32,50,51].

Interestingly, coronary artery abnormalities are more common in male MIS-C patients than in females, and also in patients with mucocutaneous and conjunctival involvement [52]. We did not find such an association between these parameters, but this may be attributed to the relatively small number of patients included in our cohort. Artery dilation is a rare complication in MIS-C, with an unclear etiology but a favorable prognosis [7,25,53], and we did not observe such. This dilation may be secondary to vasculitis or generalized hyperinflammation and cytokines storms [21].

Mannarino et al. reported data from their single-center Italian study, estimating cardiovascular involvement in 81% of MIS-C patients. The authors found two distinct groups of patients based on the ejection fraction, LVEF < 45% and >45%, which differed significantly in their clinical presentation of laboratory markers [54]. In contrast to our findings, they demonstrated ECG abnormalities in 44% of the patients and rhythm alterations in 9%.

In line with this is the retrospective multicenter cohort study of Kostik et al. (2022), who divided MIS-C patients into smaller groups based on their main symptoms. For example, patients with solely coronary artery lesions (resembling KD with younger age, thrombocytosis, and normal ferritin) and patients with solely myocardial involvement (older age, elevated ferritin, LDH, D-dimer, along with thrombocytopenia) [55]. The authors also suggested criteria in routine diagnostic procedures to confirm myocardial involvement in MIS-C (major criterion: high troponin or at least two minor criteria, e.g., swelling of the face, elevated D-dimer levels).

Similar to these results are those presented by Ludwikowska et al. (2023), who estimated that 91.5% of MIS-C patients present with cardiac involvement, including valvular insufficiency (48.2%), contractility abnormalities (41%), and a decreased left ventricular ejection fraction <55% (35.6%). The authors, however, suggested that the incidence of ventricular dysfunction might be underestimated [56]. The authors also demonstrated some associations between echo abnormalities, neurological symptoms (headache, agitation, lethargy), and breathing difficulties, usually combined with high levels of troponin and procalcitonin. Valvular insufficiency was found in 48% of the children (mitral insufficiency in 43% of the children), and tricuspid insufficiency was found in 19% of the children [56]. In contrast, we found that 5% and 9% of our subjects were affected, respectively, which is a much lower rate than that reported by Ludwikowska. The researchers also demonstrated predictive factors for cardiovascular involvement in MIS-C, including a younger age, conjunctivitis, arthritis, lymphocytosis, and higher platelet levels at admission. However, we did not find such associations.

Karagözlü et al. (2023) also demonstrated cardiovascular manifestations in MIS-C patients, especially those evaluated using MRI. The authors demonstrated ECG abnormalities in 77% of patients and echocardiographic abnormalities in 70% of patients, as well as left ventricular systolic dysfunction (45%), and pericardial effusion (32%) [57]. These results demonstrated higher rates of these cardiovascular manifestations in MIS-C than in our study. As we speculated above, the main reason for this could be that these MIS-C patients were not diagnosed with MIS-C. Karagözlü et al. also conducted follow-up examinations on the children, revealing normal CMR in all cases, with cardiac abnormalities remaining only in two children [57]. We performed follow-ups of the patients after their hospital discharge. However, most were followed up at other facilities specialized in cardiovascular diseases. Likely due to the lack of a united registry and protocol for ambulatory management of these patients, we did not manage to follow up on all of them.

Finally, a systematic review and meta-analysis performed by Yasuhara et al. revealed the longitudinal cardiac outcomes in MIS-C patients based on their mid-term (>3 months) follow-ups. The authors emphasized the importance of follow-up with these patients, observing a decrease in the rate of LV systolic dysfunction (from 46.8% at admission to 1.7% after 3 months and 2.1% after 6 months), coronary abnormalities (from 23.7% to 4.7% and 5.2%, in 3 and 6 months, respectively), and mitral regurgitation (from 56.6% to 7.5% at the 6th month) [58].

Along with the strengths of our study, including presenting data on cardiovascular manifestations in MIS-C Bulgarian children and clustering the MIS-C patients to evaluate the most common factors, our study has some limitations. First, we had a relatively small number of children with full echocardiographic descriptions, and some ECG and MRI data inconsistencies did not allow us to include all these data in the clustering analysis. We have no follow-up data, since its length varies based on clinical indications, not study aims. However, we presented cross-sectional observational data of many clinical variables related to cardiac involvement in MIS-C.

## 5. Conclusions

MIS-C is a newly identified medical condition that arises in the context of SARS-CoV-2 infection in children. As the epidemiological dynamics of the infection and the SARS-CoV2 mutations evolve, it becomes increasingly challenging to differentiate MIS-C cases and establish the link between recent infections and potential MIS-C symptoms. Since many children with MIS-C may not show cardiovascular symptoms, when their IL-6 levels are higher, these children should be screened for cardiac involvement. Based on our results, we recommend that oligosymptomatic MIS-C patients or those suspected of long COVID-19, with elevated IL-6 and other inflammatory activity, should be screened for possible cardiological involvement.

## Figures and Tables

**Table 1:** Signs of cardiovascular involvement in the MIS-C cohort of patients (* n = 36, ** n = 51, *** n = 42).

Demographic Characteristics	N (%)
**All subjects**	N = 51
Sex, male n (%)	37 (73)
Average age, years	7.52 ± 2.30
Age ≥ 5 years old, n (%)	40 (78)
Positive epidemiological history of COVID-19, n (%)	21 (41)
**General symptoms**	N, %
Fever ≥ 38 °C **	51/51 (100%)
Muscle pain/cramps **	16/51 (31%)
**Heart involvement**	N, %
Arterial hypotension **	16/51 (44%)
ECG abnormalities **	1/51 (2%)
Palpitations or precordial pain **	0/51 (0%)
Heart failure **	3/51 (8%)
Pericardial effusion ***	16/42 (38%)
Myocardial dysfunction *	15/36 (42%)
Pericarditis without effusion *	14/36 (39%)
Myocarditis *	6/36 (17%)
Coronaritis *	4/36 (11%)

ECG—electrocardiogram.

**Table 2:** Cardiovascular involvement in female and male MIS-C study subjects.

	Females	Males	Proportion Z Test *p* Values
	Percentage of Females	Percentage of Males	
Low Blood Pressure	29%	38%	0.53
High Blood Pressure	0%	21%	0.58
Pericarditis	100%	61%	0.015 *
Myocarditis	18%	13%	0.7
Heart failure	64%	49%	0.32
Myocardial dysfunction	50%	15%	0.32
Pericardial effusion	72%	29%	0.015 *
Septal dyskynesia	33%	41%	0.74
Heart wall dyskynesia	33%	32%	0.94

* Statistical significant value.

**Table 3:** Echocardiographic parameters in the MIS-C cohort of patients. The reference range is normalized to the total body surface.

ECHO Parameter	Mean, SD	Reference Range
LVDd, mm	42 ± 12	N/A
LVDd, mm/m^2^	30 ± 11.4	23–32
LVDs, mm	36.5 ± 5	N/A
LVDs, mm/m^2^	25 ± 2	
LVSF, %	34 ± 9.1	N/A
LVEF, %	62.4 ± 14.2	>55%
EDV mL	69 ± 15	N/A
EDV mL/m^2^	37.2 ± 3.7	35–75
ESV mL	27.1 ± 7.6	N/A
ESV mL/m^2^	59.8 ± 13.1	12–30
Mitral insufficiency	5% (N = 3)	N/A
Tricuspid insufficiency	9% (N = 5)	N/A

LVDd—left ventricular diameter in diastole; LVDs—left ventricular diameter in systole; LVSF—left ventricular shortening fraction; LVEF—left ventricular ejection fraction; EDV—end-diastolic volume; ESV—end-systolic volume.

**Table 4:** Laboratory parameters in the two obtained clusters from the hierarchical clustering analysis using the “Ward” method and K-means analysis on each patient’s score in the first two components of the kernel principal component analysis.

Laboratory Tests	Cluster 1	Cluster 2	*p* Value
IL-6	66.9 ± 22.5	158.4 ± 36	0.04 *
C-reactive protein	17.4 ± 2.5	21.8 ± 2.4	0.22
Procalcitonin	6.2 ± 2.2	10.2 ± 2.7	0.14
D-dimer	1686 ± 425	2528 ± 392	0.183
Ferritin	489 ± 91	598 ± 89	0.4
Fibrinogen	4.62 ± 0.3	5.7 ± 0.3	0.03 *
LDH	357 ± 47	360 ± 35	0.9
WBC count	12.7 ± 1	13.5 ± 1.5	0.69
Neutrophil count	10 ± 0.9	11 ± 1.2	0.4
hs-Troponin I	43 ± 15	119 ± 43	0.3
Creatinine kinase-MB	100 ± 28	361 ± 209	0.36
Platelets	197 ± 23	216 ± 22.5	0.56
Albumin	31.9 ± 2.2	29 ± 0.8	0.34
Creatinine	58 ± 4	69 ± 7.2	0.24

IL-6—interleukin 6; LDH—lactate dehydrogenase; hs-Troponin I—high-sensitivity Troponin I. * Statistical significant value.

## Data Availability

The data presented in this study are available from the corresponding author upon request. The data are not publicly available due to restrictions, e.g., privacy or ethics.
